# Severe malaria and death risk factors among children under 5 years at Jason Sendwe Hospital in Democratic Republic of Congo

**DOI:** 10.11604/pamj.2018.29.184.15235

**Published:** 2018-04-02

**Authors:** Augustin Mulangu Mutombo, Olivier Mukuku, Kristel Nzeba Tshibanda, Edouard Kawawa Swana, Eric Mukomena, Dieudonné Tshikwej Ngwej, Oscar Numbi Luboya, Jean-Baptiste Kakoma, Stanislas Okitotsho Wembonyama, Jean-Pierre Van Geertruyden, Pascal Lutumba

**Affiliations:** 1 Département de Pédiatrie, Faculté de Médecine, Université de Lubumbashi, Lubumbashi, Democratic Republic of Congo; 2 Institut Supérieur des Techniques Médicales de Lubumbashi, Lubumbashi, Democratic Republic of Congo; 3 Département de Santé Publique, Faculté de Médecine, Université de Lubumbashi, Lubumbashi, Democratic Republic of Congo; 4 Département de Gynécologie-Obstétrique, Faculté de Médecine, Université de Lubumbashi, Lubumbashi, Democratic Republic of Congo; 5 Faculty of Medicine & Health Sciences, University of Antwerp, Belgium

**Keywords:** Severe malaria, mortality, risk factors, children, Lubumbashi

## Abstract

**Introduction:**

Malaria is still a major public health concern in the Democratic Republic of Congo. Its morbidity and mortality challenge the actual strategies of the fight agains malaria. This study was aimed to describe the epidemiology, the clinical caracteristics and the risk factors of death associated to severe malaria in the pediatric population under 5 years at Sendwe Hospital of Lubumbashi.

**Methods:**

This analytical retrospective study was conducted in Lubumbashi, in the province of Haut-Katanga. All patients under 5 years hospitalized for severe malaria were registered from January 2014 to December 2016.

**Results:**

Among the 3,092 patients hospitalised during our study period, 452 (14.6%) were admitted for severe malaria. The average age was 27.04 months, the male sex was the most affected (53.54% with the sex-ratio 1.15). The most frequent forms of gravity noticed were cerebral malaria (48.23%) and severe anemia (46.90%). Death was noted in the evolution in 28.32%. Repeated convulsion (OR = 2.27; 95% CI: 1.47-3.48), coma (OR = 3.55; 95% CI: 2.19-5.74) and severe acute malnutrition (OR = 3.32; 95% CI: 1.56-7.06) were asscociated with a high risk of death.

**Conclusion:**

This research shows that severe malaria is still an important cause of morbidity and mortality among young children in Lubumbashi. Neurologic and anemic forms are the most frequent. The predictive signs of death are: repeated convulsions, coma and severe acute malnutrition.

## Introduction

Children under 5 years are of the most vulnerable group affected by malaria. Following the recent estimation of the World Health Organization (WHO), about 429,000 death linked to malaria were registered all over the world in 2015 and 70% of this number concerned children under 5 years [1]. *Plasmodium falciparum* malaria has a rapid evolution from a non-complicated febrile disease generally treated with an oral drug, to a potentially deathly multisystemic disease. The mortality risk associated with non complicated falciparum malaria is estimated to less than 0.1% reaching 1% when treatment fail in the context of antimalaria drugs resistance [2]. Mortality due to severe malaria among young children is generaly above 10% and increases with age [3]. Many factors predictive of death in severe malaria in children have been identified. Among them: coma and seizures [3-7], acidosis [3,4,8], respiratory distress [5, 6, 8, 9] and hypoglycemia [5,6,9]. The outcome of severe malaria depend on the nature and degree of vital orgarns dysfonction. They are different from adults to children. For exemple, severe anemia is a frequent sign of severe malaria among young children in areas of high level transmission, but it is relatively inhabitual in adults. In the Democratic Republic of Congo (DRC), malaria is a major public health problem with a morbidity and mortality challenging the actual strategies of the fight agains malaria. The actions put in place by the gouvernement consisted of introducing the artemisinin combination therapy (ACT), the distribution of bed-nets with long duration insecticide action and the free treatement of malaria for children from 0-15 years. Despite theses actions, the health demographic inquiry in Congo for the year 2014 stated that among children from 6 to 59 months, 31% were tested positive by the rapid diagnostic test of malaria and 23% by the thick blood film [10]. Following the same investigation, malaria is counted among the 3 first causes of mortality among children under 5 years and pregnant women [10]. It is within this framework that this study is inscribe. The aims is to describe the socio demographic and clinical caracteristics of malaria and to identify the death risk factors within the pediatric population under 5 years in Sendwe Hospital at Lubumbashi in DRC.

## Methods

This research was carried at Jason Sendwe Hospital, in Lubumbashi city (DRC) from the 1^st^ January 2014 to the 31 December 2016. It’s a cross-sectional study concerning children under 5 years admited for severe malaria in the pediatric emergency of this hospital. This health structure offers health cares to a large range of the population of Lubumbashi, with easy accessibility to patients from all areas. Also, chilren can have acces to a specific conslutation of a pediatrician. Children were recruted in the pediatric service. Inclusion citeria were: age under 5 years, having a positive thick blood film test to plasmodium falciparum and showing or having shown clinical signs of severe malaria according with the WHO criteria [5,11]. Children with hematuria or an urinary infection were dismissed from the study. Severe malaria has been defined as any situation including fever persisting within 48 hours, positive (thick blood film test and thine blood test) to plasmodium falciparum and at least one sign of gravity among the criteria of malaria [5,11]. Cerebral malaria has been noted on any child with a coma or a neurologic disorders persisting within 30 minutes at least without any sedative medication or any child suffering from seizure, the presence or absence of neurosensorial or psychiatric affections signs with a normal cerebrospinal liquid.

Severe anemia form was revealed through a pallid state of the patient with or without any sign of cardiac decompensation and presenting a hemoglobin rate = 5g/dl. The hemoglobinuria form has been defined as the presence of the hemoglobine in urine after a massive intravascular hemolysis (intense pallor, icterus, highly darck colored urine “coca cola”). The massive presence of hemoglobin in urine has been defined by a redish or dark aspect or urines. Microscopic hemoglobin is defined as the presence of hemoglobin in urines revealed through urinary bandelet and the presence of red blood cells observed on the microscopic analysis of urines was defined as hematury. Acute renal failure was defined by the dysfonction of renal physiology with an absolute increase of creatinine superior or equal to 0.3mg/dl, a percentage increase in serous creatinine superior or equal to 50% associated or not with oliguria. Oliguria has been defined as a low production of urine documented as inferior to 12 ml/kg per 24 hours. The data were entered into an electronic database using Microsoft Excel 2013. The risk of death during malaria treatment was measured. Patient characteristics were summarized using descriptive statistics. For the investigation of risk factors for death, we used the chi-square *test* of Yates or Fisher’s exact test to produce odds ratios (OR) and their confidence intervals at 95%. The level of statistical significance was set at 5%. All statistical analysis were performed using Epi Info software version 7.2.

## Results

**Figure 1:**
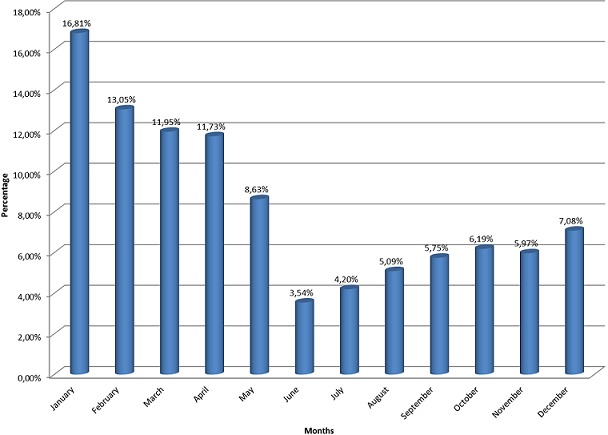
Dispatching cases of severe malaria following the month of admission

**Socio-demographic characteristics:** On the 3092 children hospitalised aged under 5 years, 452 (14.60%) were diagnosed of severe malaria. It included 242 boys (53.54%) and 210 girls (46.46%). The average age was 27.04 months (extrems: 3 months and 59 months) and 226 (50.00%) and most cases were less than 2 years (Table 1). An antimalaria treatement was administered to 161 (35.62%) before hospitalisation. The drugs given were quinine with 124 cases (77.02%) and derivatives of artemisine with 37 cases (22.98%). The month of January registered most of cases (76/452) (Figure 1).

**Table 1:** Socio-demographic characteristics and clinical forms of malaria

Variable	Frequency(n= 452)	Percentage
**Age**		
<12 months	117	25.88
12 - 23 months	109	24.12
24 - 35 months	64	14.16
36 - 47 months	48	10.62
48 - 59 months	114	25.22
Average (DS)	27.03	(18.06)
**Sex**		
Male	242	53.54
Female	210	46.46
**Clinical signs**		
Fever	434	96.02
Asthenia	271	59.96
Pallor	245	54.20
Repeated convulsions	216	47.79
Vomiting	154	34.07
Splenomegaly	149	32.96
Diarrhea	101	22.35
Coma	97	21.46
Jaundice	52	11.50
Urines dark	37	8.19
SAM	33	7.30
**Severity criteria**		
Cerebral malaria	218	48.23
Severe anemia	212	46.90
respiratory distress syndrome	41	9.07
Hemoglobinuria	35	7.74
Circulation failure	21	4.65
Hyperparasitemia	15	3.32
Hypoglycemia	8	1.77
Renal failure	3	0.66
Abnormal bleeding	3	0.66
Prostration	2	0.44

**Clinical and biological characteristics:** Signs observed on the 452 children during consultation were: fever (96.02%), asthenia (59.96%), pallor (54.20%), repeated convulsions (47.79%), vomits (34.07%), splenomegalia (32.96%), diarrhea (22.35%), coma (21.46%) and jaundice (11.50%), severe acute malnutrition was detected on 33 children (7.30%). Urines were of dark coloration in 8.19% of cases. The average rate of haemoglobin was 7.74 mg/dl (extrems: 2.90 and 16.80 mg/dl). A rate of heamoglobin inferior to 5 mg/dl was noted in 212 patients (46.90%) and glycemia inferior to 50 mg/dl noted in 8 (1.77%) creatinemia was superior to the normal rate in 3 children (0.66%).

**Severity criteria:** Severity criteria identified in the 452 were: cerebral malaria (48.23%), severe anemia (46.90%), respiratory distress syndrome (9.07%), hemoglobinuria (7.74%), circulation failure (4.65%), hyperparasitemia (3.32%), hypoglycemia (1.77%), renal failure (0.66%), abnormal bleeding (0.66%), and prostration (0.44%) (Table 1).

**Clinical forms:** Table 1 shows that the neurological and anemic forms where respectlively diagnosed in 147 cases (32.52%) and 148 cases (32.74%). These two forms where associated to each other in 36 cases (7.96%) and to the other forms in 5 cases (1.11%). The treament wase made of quinine only on 225 children (49.78%), of artemether only on 142 children (31.42%) and the combinaison of these antimalaria drug in 85 children (18.81%). This antimalaria treatement was associated to blood transfusion on 168 patients (37.17%). The average time of hospitalization was 5.1 days (extrems: 1 and 32 days). There was a favourable evolution in 281 children (62.17%); death occurred on 128 children (28.32%), the evolution was unknown for 43 children (9.51%) they left the hospital without any medical consent and before the end of the treatement.

**Predictive signs of death:** The predictive factors of death were: repeated convulsion (OR = 2.27; 95% CI: 1.47-3.48), coma (OR = 3.55; 95% CI: 2.19-5.74) and severe acute malnutrition (OR = 3.32; 95% CI: 1.56-7.06) (Table 2).

**Table 2:** Death predictive factors among children under 5 years suffering from severe malaria at the sendwe hospital in Lubumbashi

Variable	Deceased(n= 128)		Survivors (n = 281)		OR [CI95%]	p
	**n**	**%**	**n**	**%**		
**Age**						
< 24 months	65	(31.40)	142	(68.60)	1.011 [0.66-1.53]	1.0000
≥ 24 months	63	(31.19)	139	(68.81)	1.00	
**Sex**						
Female	66	(34.38)	126	(65.63)	1.31 [0.86-1.99]	0.2475
Male	62	(28.57)	155	(71.43)	1.00	
**Self-medication with antimalarials**						
Yes	47	(32.19)	99	(67.81)	1.06 [0.69-1.65]	0.8572
No	81	(30.80)	182	(69.20)	1.00	
**Season**						
Rainy	93	(34.19)	179	(65.81)	1.51 [0.96-2.39]	0.0956
Dry	35	(25.55)	102	(74.45)	1.00	
**Fever**						
Present	124	(31.63)	268	(68.37)	2.00 [0.56-7.16]	0.4148
Absent	4	(23.53)	13	(76.47)	1.00	
**Vomiting**						
Present	40	(28.17)	102	(71.83)	0.79 [0.51-1.24]	0.3774
Absent	88	(32.96)	179	(67.04)	1.00	
**Coma**						
Present	50	(53.76)	43	(46.24)	3.55 [2.19-5.74]	< 0.00001
Absent	78	(24.68)	238	(75.32)	1.00	
**Repeated convulsions**						
Present	80	(40.20)	119	(59.80)	2.27 [1.47-3.48]	0.0002
Absent	48	(22.86)	162	(77.14)	1.00	
**Pallor**						
Present	19	(39.58)	29	(60.42)	1.51 [0.81-2.81]	0.249
Absent	109	(30.19)	252	(69.81)	1.00	
**Circulatory collapse**						
Present	5	(23.81)	16	(76.19)	0.67 [0.24-1.87]	0.6044
Absent	123	(31.70)	265	(68.30)	1.00	
**Hemoglobinuria**						
Present	6	(19.35)	25	(80.65)	0.50 [0.20-1.26]	0.1970
Absent	122	(32.28)	256	(67.72)	1.00	
**SAM**						
Present	19	(61.29)	12	(38.71)	3.32 [1.56-7.06]	0.0021
Absent	109	(28.84)	269	(71.16)	1.00	
**Splenomegaly**						
Present	40	(28.99)	98	(71.01)	0.84 [0.54-1.32]	0.5443
Absent	88	(32.47)	183	(67.53)	1.00	
**Severe anemia**						
Present	50	(27.17)	134	(72.83)	0.70 [0.46-1.07]	0.1288
Absent	78	(34.67)	147	(65.33)	1.00	
**Hyperparasitemia**						
Present	2	(13.33)	13	(86.67)	0.32 [0.07-1.47]	0.2131
Absent	126	(31.98)	268	(68.02)	1.00	
**Renal failure**						
Present	2	(66.67)	1	(33.33)	4.44 [0.40-49.46]	0.4831
Absent	126	(31.03)	280	(68.97)	1.00	
**Hypoglycemia**						
Present	0	(0.00)	7	(100.00)	0.00 [0.00-1.51]	0.1039
Absent	128	(31.84)	274	(68.16)	1.00	

SAM: Severe acute malnutrition

## Discussion

Severe malaria represented 14.6% of hospitalisation during our research period. The hospitalisation frequency of children under 5 years changes following the autor, the country, the region and the period. Its variates from 13.3 to 34% in african country where we find a stable transmission rate of malaria [11-15]. The variation of frequencies can be explained by the sampling, the degree of endemic exposition, the types of studies, the quality of human ressource and the laboratory equipement in the hospitals and countries choosen.

Severe malaria mostly affects children under 5 years, this age group matches with the period when a child has lost maternal antibodies and is gradually bulding a partial immunity against malaria [16,17]. The sex ratio M/F superior to 1 in our distribution is also correlated to the tendency registered in many african countries [15,18,19]. The highest prevalence occurred during the rain season (between October and april). The tendency observed shows that the multiplication of vectors after the rain season is the cause of the resurgence of malaria cases sustainning previous studies carried out [9,20]. In malaria endemic arears, the morbidity and mortality linked to malaria occure during seasons of high transmission. Then an intermittent preventive treatement in the fight agains malaria during that intense period could have an impact in reducing the weigt of the disease as well as the risk of severe malaria [21]. The high rate of anemic and neurologic forms is correlated to studies carried out in africa. The other gravity clinical signs of the WHO where so rare in our distribution. This result joins other studies realised in the pediatric environment confirming their rarity in Africa [5,9,14,22]. The letality rate of severe malaria during this study is 28.32%. This rate variates following the autors. Our rate appears superior compared to those of previous studies realised in Lubumbashi (12.5%) for Wembonyama [23] and 8.6% for Mutombo [14]. It is also superior to those registered by other congolese and africans: Mulumba (17%) in Kinshasa (DRC) [24], Asse (13%) at Bouake (Ivory Coast) [25] and Bobossi-Serengbe (18%) at Bouar (Central African Republic) [26].

While tracing out the determinants of this high mortality, we found out that automedication to antimalaria drugs before hospitalisation was not associated to any significant high risk of death in this study. Even when used in low doses, automedication could limit a fatal out come of severe malaria [27,28]. It is a common practise noted in urban areas contributing to community participation in reducing mortality due to malaria. A study by Mutombo has shown that the perception of home-managed malaria in children from 0-5 years in Lubumbashi (DRC) was not in line with the national recommandations [29]. This imply the need to reinforcing interventions of community perspectives by assuring the training of community facilitator and the supply of artemisinine combined treatement.

The association between cerebral malaria and the death of children affected by severe malaria as registered in our distribution coinside with results from previous research carried out in Africa [6,18,30,31]. Litterature revue shows that the gravity of cerebral malaria could be explained by the sequestration of invaded red blood cell in a tissus, causing vital dysfonction in the concerned organ for exemple the production of cytokines (TNF, IFN) which may cause the release of carbon monoxyde [32-35]. Which itself causes a cerebral vasodilatation leading to an intra-cranial hypertension linked to cerebral malaria. The massive presence of parasitized red blood cells in a tissu causes tissu and metabolic hypoxia such as hypoglycemia and lactic acidosis [32,33]. This red blood cell sequestration should be responsible of the failure of the hemato-encephalic barrier with vasogenic oedema in cerebral malaria [35]. A research carried out by Pougpouratn in Thailande and in Vietnam demonstrated that death linked cerebral malaria was directed associated with massive sequestration of parasitized red blood cells in the cerebral microvessels [36]. The number of sequestered erythrocytes was correlated with pre-mortem coma, which could be explained by the fact that cerebral malaria is most often associated with a high cytoadherence of parasitized erythrocytes in the cerebral micro-vessels [36].

Like in previous studies carried out [12,27,28,37], this research shows that severe acute malnutrition was significally and independently associated to death risk during malaria. This result confirms the idea sustaining that malnutrition stands as a base to pediatric tropical pathology. There often exist a risk cumulation since in these contries, malnutrition is frequently associted to anemia and digestive parasitolosis [27,37]. The reduction of malaria letalily requires a better health care management of the pathologies or the reduction of their transmission. Although severe anemia is the first course of morbidity, it has been found no significant association between death and severe anemia. This goes the same for many other studies carried out on the african continent and above [5,19,38,39]. This could simply be due to the availability of blood products and a better understanding of its pathogeny. To our knowledge, no developed scientific study on the risk factors of death related to malaria is available.

## Conclusion

This research shows that severe malaria is still an important cause of morbidity and mortality among young children in Lubumbashi. Neurologic and anemic forms are the most frequent. The predictive signs of death are: repeated convulsions, coma and severe acute malnutrition. Further studies will be important to assess strategies for the disease control.

### What is known about this topic

- In the DRC, malaria is a major public health problem with an important rate of morbidity and mortality among children;
- Mortality due to severe malaria on young children is generally over 10% and increases with age.

### What this study adds

- In our environment, the risk factors of death for severe malaria on children under 5 years are: repeated convulsions, coma and severe acute malnutrition.
